# Inhibition of Fibrinolysis by Coagulation Factor XIII

**DOI:** 10.1155/2017/1209676

**Published:** 2017-07-06

**Authors:** Dingeman C. Rijken, Shirley Uitte de Willige

**Affiliations:** Department of Hematology, Erasmus University Medical Center, Rotterdam, Netherlands

## Abstract

The inhibitory effect of coagulation factor XIII (FXIII) on fibrinolysis has been studied for at least 50 years. Our insight into the underlying mechanisms has improved considerably, aided in particular by the discovery that activated FXIII cross-links *α*2-antiplasmin (*α*2AP) to fibrin. In this review, the most important effects of different cross-linking reactions on fibrinolysis are summarized. A distinction is made between fibrin-fibrin cross-links studied in purified systems and fibrin-*α*2AP cross-links studied in plasma or whole blood systems. While the formation of *γ* chain dimers in fibrin does not affect clot lysis, the formation of *α* chain polymers has a weak inhibitory effect. Only strong cross-linking of fibrin, associated with high molecular weight *α* chain polymers and/or *γ* chain multimers, results in a moderate inhibition fibrinolysis. The formation of fibrin-*α*2AP cross-links has only a weak effect on clot lysis, but this effect becomes strong when clot retraction occurs. Under these conditions, FXIII prevents *α*2AP being expelled from the clot and makes the clot relatively resistant to degradation by plasmin.

## 1. Introduction

Coagulation factor XIII (FXIII) is activated by thrombin into activated FXIII (FXIIIa). FXIIIa is a transglutaminase that catalyzes the formation of isopeptide bonds between the free amine group of a lysine residue and the acyl group at the end of the side chain of a glutamine residue [1]. FXIII was formerly named the fibrin-stabilizing factor, as the formation of the isopeptide bonds in fibrin results in the stabilization of fibrin. Cross-linking makes fibrin clots insoluble in weak acid or urea [2] and strongly modifies the physical properties such as clot stiffness [3]. In addition, cross-linking of fibrin makes the clot more resistant to proteolytic degradation by the fibrinolytic system.

Inherited FXIII deficiency with undetectable FXIII activity is associated with a severe bleeding tendency. Umbilical bleeding a few days after birth is a characteristic feature and occurs in about 80% of the cases, while intracranial hemorrhage at birth constitutes a main threat to life [4]. It is not fully established which mechanisms are primarily responsible for the bleeding tendency. These mechanisms may involve the lack of physical stabilization of fibrin, the enhanced sensitivity to fibrinolysis, and/or even other mechanisms.

In this paper, we will review the effect of FXIII on fibrinolysis, in particular on the extent of inhibition of lysis and the biochemical mechanisms that are involved. There has been a long-running debate on these topics with many apparently conflicting reports. While reviewing the literature, it is helpful to distinguish mechanisms that play a role in purified fibrin clots and mechanisms that play a role in plasma or whole blood clots where cross-linking of other proteins could occur. This paper shows that most important mechanisms are gradually being elucidated. Excellent reviews about FXIII, including its effect on fibrinolysis, have been published earlier [1, 5].

## 2. The Fibrinolytic System

The conversion of the inactive proenzyme plasminogen into the active enzyme plasmin is the central step in the fibrinolytic system [6]. Plasmin degrades fibrin into soluble fibrin degradation products. Two physiologic plasminogen activators are capable of catalyzing the conversion of plasminogen: tissue-type and urokinase-type plasminogen activator (t-PA and u-PA, resp.). The most important plasminogen activator for fibrin degradation is probably t-PA, although u-PA may also be involved in a complementary action of t-PA and u-PA [7]. In the absence of fibrin, t-PA is a poor plasminogen activator. However, in the presence of fibrin, the plasminogen activator activity of t-PA is two orders of magnitude higher as a result of the binding of both t-PA and plasminogen to fibrin and the formation of a cyclic ternary complex. This makes t-PA a fibrin-specific agent.

Inhibition of the fibrinolytic system may occur at the level of plasminogen activation, mainly by plasminogen activator inhibitor-1 (PAI-1) [8]. The two chain forms of t-PA and u-PA are also efficiently inhibited by plasminogen activator inhibitor-2 (PAI-2), but the plasma levels of PAI-2 are normally low or even undetectable (except during pregnancy), and other intracellular functions of PAI-2 have been postulated [9]. Inhibition of the fibrinolytic system may also occur at the level of plasmin, mainly by *α*2-antiplasmin (*α*2AP) [10]. PAI-1 and PAI-2, as well as *α*2AP, are members of the serine protease inhibitor (serpin) superfamily. Fibrin degradation is additionally inhibited by thrombin-activatable fibrinolysis inhibitor (TAFI) [11]. TAFI represents a link between coagulation and fibrinolysis. The inhibitor is slowly activated by thrombin, but this activity of thrombin is three orders of magnitude higher in the presence of thrombomodulin. The activated form (TAFIa) is a plasma carboxypeptidase B and has a short half-life of about 10 min under physiologic conditions. The antifibrinolytic activity of TAFIa is based on the elimination of C-terminal lysine and arginine residues from partially degraded fibrin. This results in a strongly reduced binding of plasminogen on partially degraded fibrin and a concomitant reduction of the activation of plasminogen [12].

Two types of lysis can be distinguished both in vivo and in vitro: external and internal lysis [13]. In internal lysis, all fibrinolytic components are homogeneously distributed within the clot, whereas in external lysis plasminogen activators, t-PA or u-PA, are acting from the outside of the clot. In in vitro experiments, internal lysis is accomplished by adding plasminogen activator to fibrinogen, plasma, or blood before clot formation is induced, while external lysis is obtained by the incubation of a preformed clot in a milieu which is enriched in a plasminogen activator.

## 3. Effect of Factor XIIIa on Lysis of Purified Fibrin Clots

Cross-linkage of fibrin by FXIIIa results in a rapid formation (within minutes under physiological conditions) of cross-links between *γ* chains yielding *γ* chain dimers and in a slower formation (minutes to hours) of cross-links between *α* chains yielding *α* chain polymers consisting of a varying number of *α* chains. *β* chains are not involved directly in the cross-linking of fibrin [14]. Only a small number of *α* chain polymers have been formed by the time all the *γ* chains have been linked, illustrating the difference in reaction rates. A minor amount of cross-linking also occurs between *α* and *γ* chains and, upon prolonged incubation (hours to days), between interfibril *γ* chains resulting in *γ* chain trimers and *γ* chain tetramers [15].

Some early fibrinolysis studies did not find an effect of FXIIIa on the lysis of purified fibrin clots [16, 17]. Francis and Marder [18], however, were able to demonstrate that the intramolecular cross-links producing *α* chain polymers, in particular the very high molecular weight *α* chain polymers obtained with elevated FXIIIa concentrations, are associated with the inhibition of fibrinolysis. In addition, Siebenlist and Mosesson [19] found inhibition of lysis by FXIIIa and claimed that resistance to fibrinolysis is not induced by *α* chain polymer formation or *γ* chain dimer formation, but by *γ* chain multimer formation. Although the latter two studies disagree about the responsible structures, they agree that inhibition of fibrinolysis occurs particularly after a very strong cross-linking of fibrin. It is quite possible that the studies that found no or only a small effect did not induce such a strong cross-linking. Indeed, recent studies demonstrating small inhibitory effects of fibrin cross-linking on fibrinolysis were not performed under strong cross-linking conditions [20]. The small inhibitory effects of the latter study were also observed with a recombinant mutant of fibrinogen (*γ*Q398N/Q399N/K406R), which does not allow for *γ* chain cross-linking, suggesting that *α* chain cross-linking explained the inhibition [21]. This is in line with the above-mentioned conclusion from Francis and Marder [18].

## 4. Effect of Factor XIIIa on Lysis of Fibrin Clots Prepared from Plasma or Whole Blood

FXIIIa does not only catalyze intramolecular cross-links within fibrin but also catalyze intermolecular cross-links between fibrin and other proteins. In a recent proteomic study, 48 proteins were found to be cross-linked to plasma clots [22]. In particular, proteins with an inhibitory effect on fibrinolysis are of importance for clot stability. These proteins include *α*2AP, PAI-2, TAFI, and complement C3. Cross-linking of *α*2AP occurs via Gln14 in *α*2AP and Lys303 in the *α* chain of fibrin [23]. Other minor cross-linking sites in *α*2AP are Gln34, Gln431, and Gln459 [24]. Cross-linking of PAI-2 occurs via Gln83 and Gln86 in PAI-2 and at one of six lysine residues in the *α* chain of fibrin, but not at Lys303 [25]. Potential cross-linking sites in TAFI are Gln2, Gln5, and Gln292 [26]. Finally, recent studies suggest that complement C3 prolongs fibrinolysis and is cross-linked to fibrin by FXIIIa [27]. The mechanism of the inhibition of fibrinolysis by complement C3 has not yet been elucidated. As described below, the cross-linking of *α*2AP has the strongest effect on fibrinolysis as compared to the cross-linking of PAI-2, TAFI, and complement C3.

## 5. Cross-Linking of ***α***2-Antiplasmin to Fibrin

An important discovery for the mechanism of the inhibition of fibrinolysis by FXIII is that FXIIIa cross-links *α*2AP to fibrin when blood is clotted in the presence of calcium ions [28]. The cross-linked *α*2AP is fully active and essential for the inhibition of fibrinolysis, particularly of spontaneous fibrinolysis by t-PA-induced plasminogen activation on the fibrin surface [29]. The cross-linking of *α*2AP to fibrin occurs rapidly; maximal *α*2AP cross-linking is almost reached when *α* chain polymerization has just started [30]. An unexplained phenomenon is that the cross-linking of *α*2AP stops at about 30% incorporation (corresponding to about one *α*2AP molecule per 25 molecules of fibrin), whereas the cross-linking of fibronectin to fibrin in the same experiments continues to about 100%. Enhancing the concentrations of FXIIIa increases the rate of *α*2AP cross-linking but does not change the maximal incorporation [30]. It has been suggested that FXIIIa not only accelerates the cross-linking of *α*2AP but also accelerates the release of cross-linked *α*2AP and that the latter activity explains the partial incorporation [31]. However, other mechanisms related to *α*2AP heterogeneity (see below) or structural hindrance by *α* chain polymerization should be considered as well, although structural hindrance does not appear to play a role in the cross-linking of *α*2AP to fibrinogen [32].

It is somewhat remarkable that higher levels of FXIII do not increase the maximal level of *α*2AP cross-linking [30], whereas the Leu variant of the Val34Leu polymorphism of FXIII, which is more rapidly activated by thrombin than the Val variant, shows a higher incorporation of *α*2AP than the Val variant. This occurs in plasma samples of healthy subjects [33] as well as in purified systems [34]. The two latter studies utilized the same microtiter plate incorporation assay. This suggests that the assay reflects the rate of *α*2AP incorporation more than the maximal incorporation.

## 6. Heterogeneity of ***α***2-Antiplasmin

In the circulation, *α*2AP undergoes both N-terminal and C-terminal proteolytic modifications that significantly modify its functional properties [10]. Approximately 35% of circulating *α*2AP lacks the C-terminus containing the binding site for plasmin(ogen) which is crucial for the rapid inhibitory mechanism of *α*2AP [35]. Although this so-called nonplasminogen-binding form of *α*2AP has not yet been purified and biochemically characterized, there is evidence that *α*2AP with an intact C-terminus, also called plasminogen-binding form, is selectively cross-linked to fibrin by FXIIIa [36].

Approximately 70% of circulating *α*2AP is cleaved at the N-terminus between Pro12 and Asn13 of full-length *α*2AP which has Met as the N-terminus (Met-*α*2AP) [37]. The 12-residue shorter form has Asn as the N-terminus (Asn-*α*2AP) and is cross-linked to fibrin ~13 times faster than Met-*α*2AP, probably because the cross-linking site Gln14 is more exposed in Asn-*α*2AP than it is in Met-*α*2AP [38]. Lysis rates of plasma clots are slower when the plasma samples contain relatively more Asn-*α*2AP [38]. The enzyme that cleaves Met-*α*2AP in vitro is named antiplasmin-cleaving enzyme (APCE) and is identical to the soluble form of membrane-bound fibroblast activation protein in the circulation (cFAP) [38]. Correlation studies of circulating cFAP levels and percentage *α*2AP N-terminal cleavage in the plasma samples of patients and healthy controls confirm that cFAP is also responsible for the cleavage in vivo [39, 40]. The *α*2AP gene codes for either Arg or Trp as the sixth amino acid. cFAP cleaves Met-*α*2AP(Arg6) ~8-fold faster than Met-*α*2AP(Trp6), suggesting that this Arg6Trp polymorphism may be functionally significant [41].

If we assume that the N-terminal and C-terminal proteolytic modifications of *α*2AP occur independently of each other, we can calculate that 65%  ×  70% = 45.5% of the *α*2AP in plasma has a form (Asn-plasminogen-binding *α*2AP) that is rapidly incorporated in fibrin by FXIIIa. This may partially explain why the maximal incorporation of *α*2AP is only ~30%.

## 7. Relative Contributions of Fibrin-Fibrin Cross-Links and Fibrin-***α***2-Antiplasmin Cross-Links to Inhibition of Lysis

During blood clotting, FXIIIa introduces cross-links within fibrin as well as between fibrin and *α*2AP. The relative contributions of the two types of cross-links in the inhibition of fibrinolysis by FXIII have been thoroughly investigated. Jansen et al. [42] studied the lysis rate of fresh whole blood clots containing t-PA that was added before clotting in vitro. They reported that fibrin-*α*2AP cross-linking explains the FXIIIa-induced resistance of blood clots to fibrinolysis, whereas fibrin-fibrin cross-linking has only a small, if any, influence. This was later confirmed by Fraser et al. [43], who showed that the antifibrinolytic function of FXIII in plasma clots prepared in a Chandler loop and incubated in a buffer containing t-PA is independent of fibrin-fibrin cross-linking and is expressed exclusively through *α*2AP. Reed and Houng [44] studied t-PA-induced fibrinolysis in anesthetized ferrets with pulmonary emboli and found, in contrast to the previous investigators, that both fibrin-fibrin and fibrin-*α*2AP cross-linking caused resistance to lysis.

Significant inhibition of fibrinolysis by fibrin-fibrin cross-links requires strong cross-linking conditions, resulting in very high molecular weight *α* chain polymers and/or *γ* chain trimers and tetramers, whereas significant inhibition of fibrinolysis by fibrin-*α*2AP cross-links occurs immediately after clotting, as these links are formed rapidly. Therefore, it can be anticipated that the relative contribution of fibrin-fibrin cross-links to the total inhibition of fibrinolysis by FXIII depends on the extent of cross-linking and increases, for instance, with the age of a thrombus.

## 8. Effect of Clot Compaction and Clot Retraction on Lysis

The inhibition of clot lysis by FXIII is determined not only by the degree of cross-linking but also by the design of the lysis experiments. This was already concluded in 1979 in a paper about the existing controversy concerning the question of whether or not FXIII cross-linking affects fibrinolytic rates [45]. In this connection, Mutch et al. [46] published an interesting observation that model thrombi formed under flow in a Chandler loop and subsequently incubated in a buffer containing t-PA reveal a significant effect of FXIII on fibrinolysis. This effect is also revealed by thrombi prepared from platelet-free plasma. It is suggested that flow is required during clot formation.

**Figure 1:**
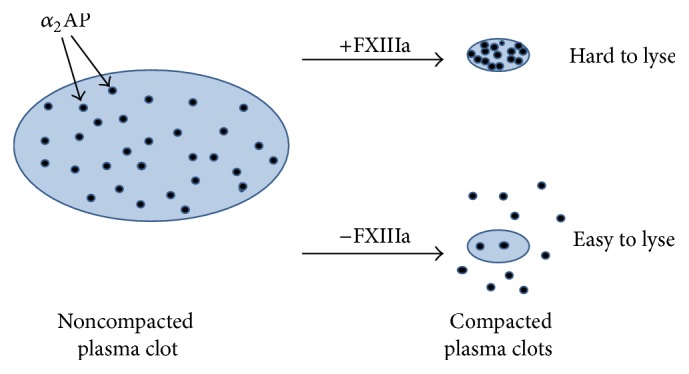
Activated factor XIII (FXIIIa) partially prevents *α*2-antiplasmin (*α*2AP) from being expelled from a plasma clot during compaction and thereby strongly inhibits fibrinolysis. Taken from Rijken et al. [47].

We recently studied the effect of clot retraction on the antifibrinolytic effect of FXIII [47], stimulated by preliminary data from Sakata and Aoki pointing to the potential importance of clot retraction [29]. Our studies started with platelet-poor and t-PA-containing plasma clots with and without physical compaction by centrifugation, as a model of platelet-mediated clot retraction. Without compaction, FXIII slightly inhibited clot lysis (1.6-fold). With compaction, however, FXIII revealed a strong inhibition of clot lysis (7.7-fold). Because FXIII did not show inhibition with either compacted or noncompacted clots prepared from *α*2AP-deficient plasma, the inhibition was completely ascribed to the cross-linking of *α*2AP, in agreement with previous studies [42, 43]. Under these conditions, the fibrin-fibrin cross-links were apparently too limited to play a significant role. The same held true for potential cross-links between fibrin and other proteins such as TAFI and PAI-2. Experiments with platelet-rich plasma clots, with and without platelet-mediated clot retraction, showed similar results as platelet-poor plasma clots with and without compaction. The slight inhibition by FXIIIa of the lysis of noncompacted or nonretracted plasma clots implied that cross-linked *α*2AP is a somewhat better inhibitor of plasmin that is generated on the fibrin surface than non-cross-linked *α*2AP. However, this type of inhibition in assays without compaction or retraction is small and probably not easily detectable since various studies in the literature report no inhibition (e.g., [45]). The extra and strong inhibition of the lysis of compacted or retracted plasma clots is easily explained, since FXIIIa partially prevents *α*2AP from being expelled from the clot during compaction or retraction. This mechanism is illustrated in Figure 1.

The requirement of clot compaction for the full expression of the antifibrinolytic effect of FXIII in clot lysis assays explains the old controversy between Gaffney and Whitaker [48] and Rampling and Flexman [45]. The first authors found a strong inhibitory effect on lysis of plasma clots harvested by winding the clot onto a glass rod (thus with compaction), whereas the latter authors found no inhibitory effect using undisturbed plasma clots (thus without compaction). The requirement of clot compaction also explains why the lysis of Chandler loop thrombi is sensitive to FXIII [46], as Chandler loop thrombi are always compacted. Therefore, compaction and not the formation under flow is the essential feature a clot must show in order to reveal FXIII-dependent clot resistance.

## 9. (Patho)physiologic Aspects

What is the (patho)physiologic meaning of the mechanism illustrated in Figure 1? It is not easy to answer this question, because our knowledge of platelet-mediated clot retraction is limited. We do not know when, where, and how fast clot retraction occurs in the body. However, it is clear from Figure 1 that in vivo retracted clots are fairly resistant to fibrinolysis owing to the cross-linking of *α*2AP by FXIIIa. Without FXIIIa, *α*2AP would be largely expelled from the clot during retraction. It is assumed that platelet-mediated clot retraction can occur in the absence of FXIIIa activity. This is still a matter of debate [49], but in our hands platelet-rich clots do retract in the presence of FXIIIa inhibitor [47]. Full absence of FXIII in patients is rare, but low levels do occur more frequently, for instance, in acquired FXIII deficiency. Studies in purified systems [50] and in platelet-poor plasma [46] indicate that plasma FXIII levels of 50% of normal are required for the optimal inhibition of fibrinolysis. FXIII levels below 50% are associated with a smaller *α*2AP incorporation and enhanced clot lysis. Platelets and platelet FXIII could contribute to the cross-linking of *α*2AP to fibrin [51–53]. Hemostasis in FXIII-deficient patients is already normalized at therapeutic FXIII plasma levels of 3–5% [4]. This suggests that the severe bleeding problems of FXIII-deficient patients are not primarily caused by enhanced clot lysis, but by impaired (physical) stability of the clots due to insufficient formation of *γ* chain dimers and possibly *α* chain polymers. Enhanced fibrinolysis could contribute, however, to the severity of the bleeding tendency, as recently observed in a subpopulation of patients with von Willebrand disease [54].

While enhanced fibrinolysis may be associated with bleeding, impaired fibrinolysis could result in a thrombotic tendency. Although the cross-linking of *α*2AP to fibrin seems already to be maximal at a plasma FXIII level of 50%, the incorporation of *α*2AP is only partial (about 30%) and one could imagine that the incorporation may increase or may become faster in certain physiologic or pathophysiological conditions. One example is the earlier-mentioned increased rate of *α*2AP incorporation by the Leu variant of the Val34Leu polymorphism of FXIII [33, 34]. Similar increases might be caused by variations in *α*2AP (for instance, in the N-terminal or C-terminal heterogeneity) or in fibrinogen.

## 10. Conclusions

The inhibitory effects of FXIII-mediated cross-links on fibrinolysis are summarized in Table 1. Fibrin-fibrin cross-links are responsible for weak effects, although very strong cross-linking conditions in vitro result in a moderate inhibition of fibrinolysis. It is not yet clear to what extent these strong cross-linking conditions occur in vivo. Fibrin-*α*2AP cross-links are also responsible for only weak effects, but these cross-links result in strong inhibition of clot lysis when clot retraction occurs. We assume that blood clotting in vivo is often followed by platelet-mediated clot retraction, suggesting that this inhibition observed in vitro is representative of the in vivo situation.

The effects summarized in Table 1 are to a great extent based on internal lysis experiments. In external lysis, fibrinolytic components are transported from the outside to the inside of the clot by diffusion and, in particular in vivo, by flow. This transport may be affected by cross-linking. Although fibrin cross-linking has only minor effects on global network structure, it has a strong effect on individual fibers since they become more compact by cross-linking [55]. This will reduce the transport of, for instance, plasminogen activators through the fibers and reduce external clot lysis.

## Figures and Tables

**Table 1:** Summary of inhibitory effects of FXIII-mediated cross-links on fibrinolysis.

Type of cross-links	Inhibitory effect
*Fibrin-fibrin cross-links*	
(i) *γ* chain dimers	None
(ii) *α* chain polymers	Weak
(iii) High molecular weight *α* chain polymers and/or *γ* chain multimers	Moderate
*Fibrin-α2-antiplasmin cross-links*	
(i) In nonretracted fibrin clots	Weak
(ii) In retracted fibrin clots	Strong
